# Dual kidney transplantation offers a safe and effective way to use kidneys from deceased donors older than 70 years

**DOI:** 10.1186/s12882-019-1664-8

**Published:** 2020-01-06

**Authors:** Kyo Won Lee, Jae Berm Park, So Ra Cha, Seo Hee Lee, Young Jae Chung, Heejin Yoo, Kyunga Kim, Sung Joo Kim

**Affiliations:** 1 Department of Surgery, Samsung Medical Center, Sungkyunkwan University School of Medicine, 81 Irwon-ro, Gangnam-gu, Seoul, 06351 South Korea; 2 Organ Transplantation Center, Samsung Medical Center, Seoul, South Korea; 3 Statistics and Data Center, Samsung Medical Center, Seoul, South Korea

**Keywords:** Expanded criteria donor, Old age donor, Dual kidney transplantation

## Abstract

**Purpose:**

Dual kidney transplantation (DKT) offers a way to extend the use of kidneys from expanded criteria donors (ECDs). Here, we compared the outcomes of DKT with those of single kidney transplantation from standard criteria donors (SCDs) and ECDs.

**Methods:**

In 2014, we began performing DKT using both kidneys from deceased donors greater than 70 years of age with one of two risk factors: serum creatinine (sCr) level over 3.0 mg/dl or eGFR under 30 ml/min. By 2017, we had performed 15 DKTs. We compared the outcomes of the 15 DKT recipients with those of 124 patients who received a kidney from an SCD and 80 patients who received a kidney from an ECD.

**Results:**

Compared with ECDs and SCDs, DKT donors were older, had a higher diabetes burden, and a higher sCr level (*p* < 0.01, < 0.01, and 0.03, respectively). DKT recipients were also older and had a higher diabetes burden than recipients of kidneys from ECDs and SCDs (*p* < 0.01, both). DKT recipients had a lower nadir sCr and shorter duration to nadir sCr than single ECD KT recipients (*p* < 0.01and 0.04, respectively).

**Conclusions:**

The survival rates of DKT grafts were compatible with those of single KT grafts. Therefore, DKT may be considered a suitable an option to expand the donor pool.

## Background

The rapidly growing incidence of chronic kidney disease and limited supply of donor organs has led to an increase in the number of patients awaiting kidney transplantation (KT). To overcome this organ shortage, kidneys from expanded criteria donors (ECD) or suboptimal donors are now widely used for transplantation. Importantly, the reported outcomes for KTs from ECDs are not inferior to those for KTs from standard criteria donors (SCDs) [1–3]. However, there remains a huge gap between the supply and demand for donor kidneys. According to data from Korean Network for Organ Sharing (KONOS), 7.9% of kidneys from potential donors were discarded between 2010 and 2018. Kidneys from donors older than 60 or 70 are more likely to be discarded (12.0 and 18.1%, respectively). These high discard rates among older donors in their 60’s and 70’s are notable, since the proportion of such donors is increasing, accounting for 24.5 and 6.7% of Korean donors, in 2018, respectively. Dual kidney transplantation (DKT) offers a way to address the shortage of donor kidneys by reducing the organ discard rate [4]. Indeed, many centers have recently started to perform DKTs using their own selection criteria [5–10]. As of 2013, deceased donors in Korea could donate both kidneys to a single candidate when they were older than 70 and met at least one of the following criteria: 1) Estimated glomerular filtration rate (eGFR) calculated by MDRD equation less than 30 mL/min without improvement, 2) Serum creatinine (sCr) level higher than 3.0 mg/dL without improvement, or when a single kidney is refused by all other centers.

Here, we describe a single center experience and outcomes of DKT using kidneys from extremely marginal donors with age over 70 and acute kidney injury.

## Methods

### Study design

This study was a retrospective single center historical cohort study and analyzed Samsung Medical Center electronic medical record and kidney transplantation database. We screened the records of 242 recipients who underwent deceased donor kidney transplantation (DDKT) between January 2014 and November 2017 in Samsung Medical Center. We excluded three pediatric recipients, four recipients who underwent *en-bloc* kidney transplantation, and 16 recipients who underwent multi-organ transplantation. The remaining 219 recipients included in the study were divided into three groups according to donor status. Group 1 (*n* = 124) consisted of patients who received a kidney from an SCD; Group 2 (*n* = 80) consisted of patients who received a kidney from an ECD; Group 3 (*n* = 15) consisted of patients who underwent DKT.

All data analyzed in the study were derived from our institution’s electronic medical records and kidney transplantation database. The institutional review board of Samsung Medical Center approved this study protocol (SMC 2018–03-035) and waived the requirement for written informed consent.

### Donor selection criteria and definitions

ECD was defined based on United Network for Organ Sharing (UNOS) criteria. ECDs consisted of deceased donors (DDs) older than 60 years and DDs 50 to 59 years of age who met two of the following criteria: (1) history of hypertension, (2) cerebrovascular accident as a cause of brain death, and (3) final pre-procurement sCr level > 1.5 mg/dL [11]. DKTs were attempted when the donor was older than 70 and met at least one of the following criteria: (1) an eGFR calculated by MDRD equation < 30 mL/min without improvement or (2) an sCr level > 3.0 mg/dL without improvement or, regardless of donor age, when the single kidney was refused by all other centers. Kidney(s) were not accepted if they were grossly discolored or atrophied, or if the donor’s sCr level had risen gradually for more than 5 days.

Urine leakage, ureteral stricture, post-operative bleeding, renal artery stenosis, and lymphocele requiring drainage were included as surgical complications. Graft failure was defined as the need for either permanent dialysis or re-transplantation. Delayed graft function (DGF) was defined as needing dialysis during the first week post-transplantation. eGFR values were calculated by the MDRD study equation.

### Surgical technique

For DKT, we used a midline incision and implanted the graft kidneys in the intraperitoneal space separately on both sides. Anastomoses to the iliac vessels were performed separately on each side. Ureteroneocystostomies were performed bilaterally after revascularization of both kidneys using the Lich-Gregoir technique, with a double J stent for each ureter.

### Postoperative management

We used rabbit anti-thymocyte globulin (rATG, 1.5 mg/kg, three doses on days 0, 1, 2) as an induction immunosuppressive agent for recipients with donor-specific anti-HLA antibodies (DSAs) or history of KT, as well as in single ECD KT and DKT cases. Otherwise, we used an interleukin-2 receptor antagonist induction (Baxilixmab, 20 mg/kg, two doses on days 0 and 4) as the induction immunosuppressive agent. A single dose of anti-CD20 monoclonal antibody (Rituximab, 375 mg/m^2^, single dose on day 0) was given to all recipients with DSAs. In all cases, the induction agent was ultimately decided according to the physician’s preference.

Tacrolimus, mycophenolate, and steroids were used as maintenance immunosuppressive agents. Our detailed protocol for maintenance immunosuppressive agents, as well as infection prophylaxis and monitoring, has been previously described [12].

Protocol biopsies were performed 14 days and one year after KT. Biopsies were not performed for DKT recipients since the grafts were not fixed in the retroperitoneal space and the risk of post-biopsy bleeding was high. Biopsies were also performed in case of suspected acute rejection, such as a noted elevation in sCr level.

### Statistical analysis

Continuous variables were analyzed with by the Kruskal Wallis test and post-hoc analysis was performed by the Wilcoxon rank sum test. Categorical variables were analyzed by Fisher’s exact test. Graft and patient survival rates were obtained by Kaplan-Meier analysis and risk factor analysis was performed by Cox proportional-hazards regression model. Stepwise selection methods were applied to identify co-variables in the Cox proportional-hazards regression model (significance criteria 0.05 for entry and removal). The Generalized Estimating Equation (GEE) was applied to analyze repeated measurements for sCr and eGFR levels. Statistical analyses were performed using SAS version 9.4 (SAS Institute Inc., Cary, NC, USA).

## Results

### Donor and recipient characteristics

Donor characteristics are summarized in Table 1. The mean donor age, BMI, and pre-retrieval sCr level of the DKT group were 74.5 yrs., 24.5 kg/m^2^, and 2.1 mg/dL, respectively which represented the highest values among groups (*p* < 0.01, 0.01, and 0.03). More donors of the ECD and DKT groups had diabetes or hypertension (*p* < 0.01, both) and died from cerebrovascular disease (*p* = 0.03). The kidney donor profile index (KDPI) and kidney donor risk index (KDRI) were highest in the DKT group (*p* < 0.01, both). Recipient characteristics are summarized in Table 2. The mean recipient age was 63.7 yrs. in the DKT group, which was the highest among the groups (*p* < 0.01). More diabetic recipients underwent DKT (*p* < 0.01). Patients with a previous history of KT, panel reactive antigen (PRA) over 50%, or DSAs were more prevalent in the SCD group (*p* = 0.02, 0.03, and 0.02, respectively). Mean cold ischemic times were not different among groups. Mean dialysis duration before KT in the DKT group (4.8 years) was shorter than in the ECD group (6.1 years), although the difference was not statistically significant (*p* = 0.17).

**Table 1:** Donor characteristics

	SCD (*n* = 124)	ECD (*n* = 80)	DKT (*n* = 15)	*p* value	SCD vs DKT	ECD vs DKT
Age (year)	42.6 ± 12.0	64.4 ± 7.2	74.5 ± 5.1	< 0.01	< 0.01	< 0.01
Sex (M:F)	78:46	51:29	7:8	0.45		
BMI (kg/m^2^)	23.5 ± 4.0	24.9 ± 3.4	24.5 ± 4.3	0.01	0.99	0.83
Pre-retrieval Serum Cr (mg/dL)	1.8 ± 1.4	2.0 ± 1.6	2.1 ± 0.7	0.03	0.03	0.13
Serum Cr > 2.0 (%)	34 (27.4)	25 (31.3)	8 (53.3)	0.13		
DM (%)	9 (7.5)	25 (33.3)	8 (57.1)	< 0.01	< 0.01	0.26
HTN (%)	18 (15.0)	40 (53.3)	6 (42.9)	< 0.01	0.04	0.99
CRRT (%)	16 (14.9)	12 (17.1)	2 (13.3)	0.95		
Cause of death				0.03	0.04	0.33
CVA	41 (33.1)	40 (50.0)	8 (53.3)			
Trauma	26 (21.0)	15 (18.8)	6 (40.0)			
Hypoxic brain damage	50 (40.3)	22 (27.5)	1 (6.7)			
unknown	7 (5.7)	3 (3.8)	0 (0)			
KDPI	52.4 ± 22.5	91.6 ± 8.4	99.6 ± 0.6	< 0.01	< 0.01	< 0.01
KDRI	1.1 ± 0.2	1.8 ± 0.4	2.5 ± 0.4	< 0.01	< 0.01	< 0.01

*BMI* body mass index, *CRRT* continuous renal replacement therapy, *CVA* cerebrovascular accident, *KDPI* kidney donor profile index, *KDRI* kidney donor risk index

**Table 2:** Recipient characteristics

	SCD (*n* = 124)	ECD (*n* = 80)	DKT (*n* = 15)	*p* value	SCD vs DKT	ECD vs DKT
Age (year)	49.3 ± 10.1	55.2 ± 10.1	63.7 ± 6.7	< 0.01	< 0.01	< 0.01
Sex (M:F)	80:44	57:23	13:2	0.19		
Dilaysis duration (yrs)	6.6 ± 4.5	6.1 ± 3.3	4.8 ± 2.5	0.17		
History of KT (%)	24 (19.4)	6 (7.5)	0 (0)	0.02	0.15	0.99
BMI	23.0 ± 3.5	23.6 ± 2.8	22.6 ± 2.9	0.06		
DM (%)	22 (17.7)	28 (35.0)	12 (80.0)	< 0.01	< 0.01	< 0.01
HTN (%)	97 (78.2)	70 (87.5)	11 (73.3)	0.16		
HLA mm ^a^	3 (0–6)	4 (0–6)	4 (2–6)	< 0.01	0.03	0.59
PRA > 50%	25 (20.8)	6 (7.5)	1 (6.7)	0.03	0.55	0.99
DSA (+)	21 (17.2)	5 (6.25)	0 (0)	0.02	0.25	0.99
CIT (min)	287.9 ± 83.6	281.7 ± 89.5	290.3 ± 107.0	0.90		
Induction agent (rATG:basiliximab:rituximab)	79:27:18	71:6:3	15:0:0	< 0.01	0.03	0.99

*KT* kidney transplantation, *BMI* body mass index, *DM* diabetes mellitus, *HTN* hypertension, *HLA* human leukocyte antigen, *PRA* panel reactive antibody, *DSA* donor specific antibody, *CIT* cold ischemic time

^a^Median (range),

### Clinical outcomes

**Fig. 1:**
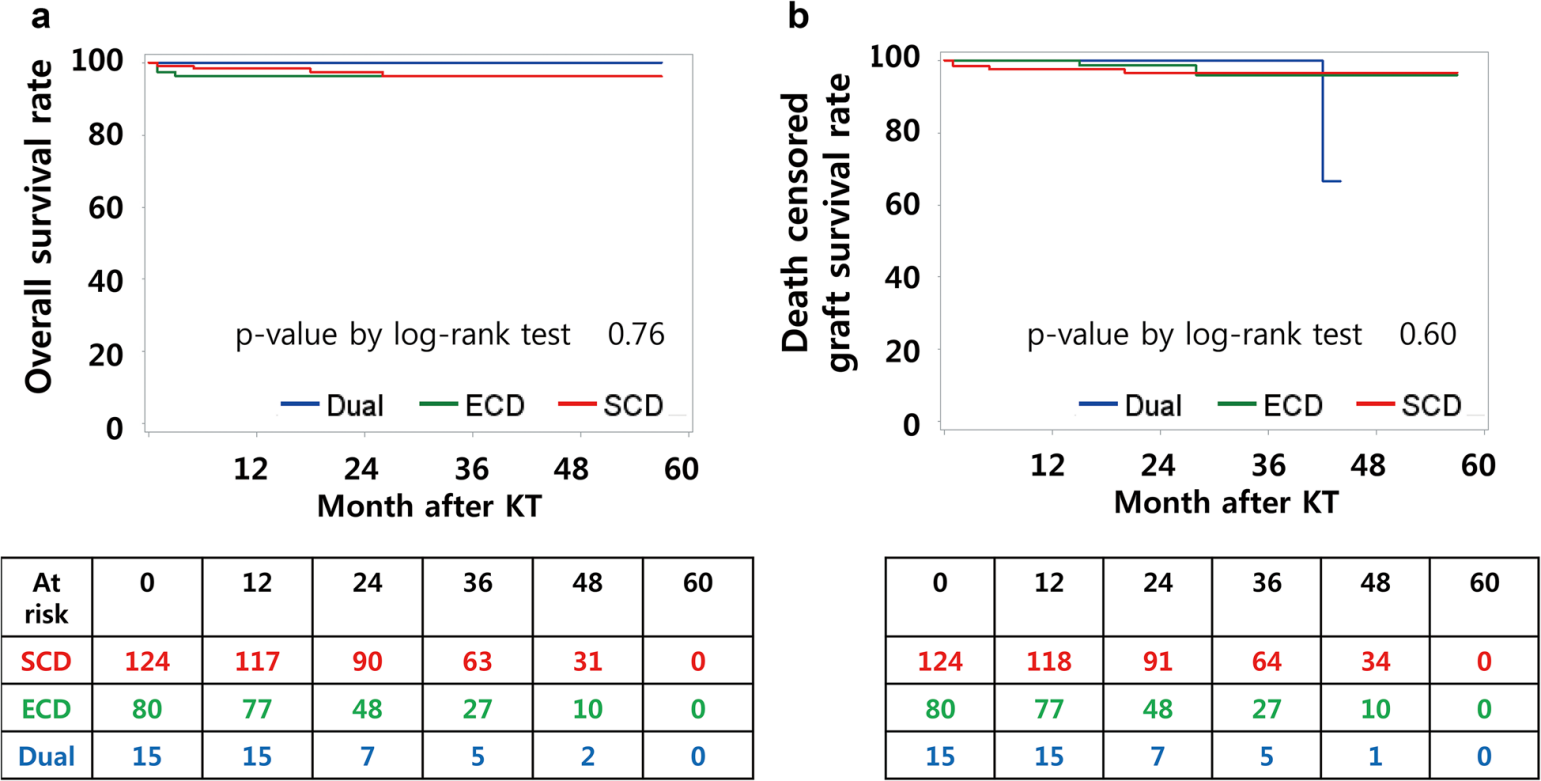
Overall survival and death censored graft survival curves. (**a**) 3 years after KT, patient survival was 96.2% in SCD group, 96.2% in ECD group, and 100% in DKT group. (**b**) Death censored graft survival at 3 years after KT was 96.6% in SCD group, 95.9% in ECD group, and 100% in DKT group

**Fig. 2:**
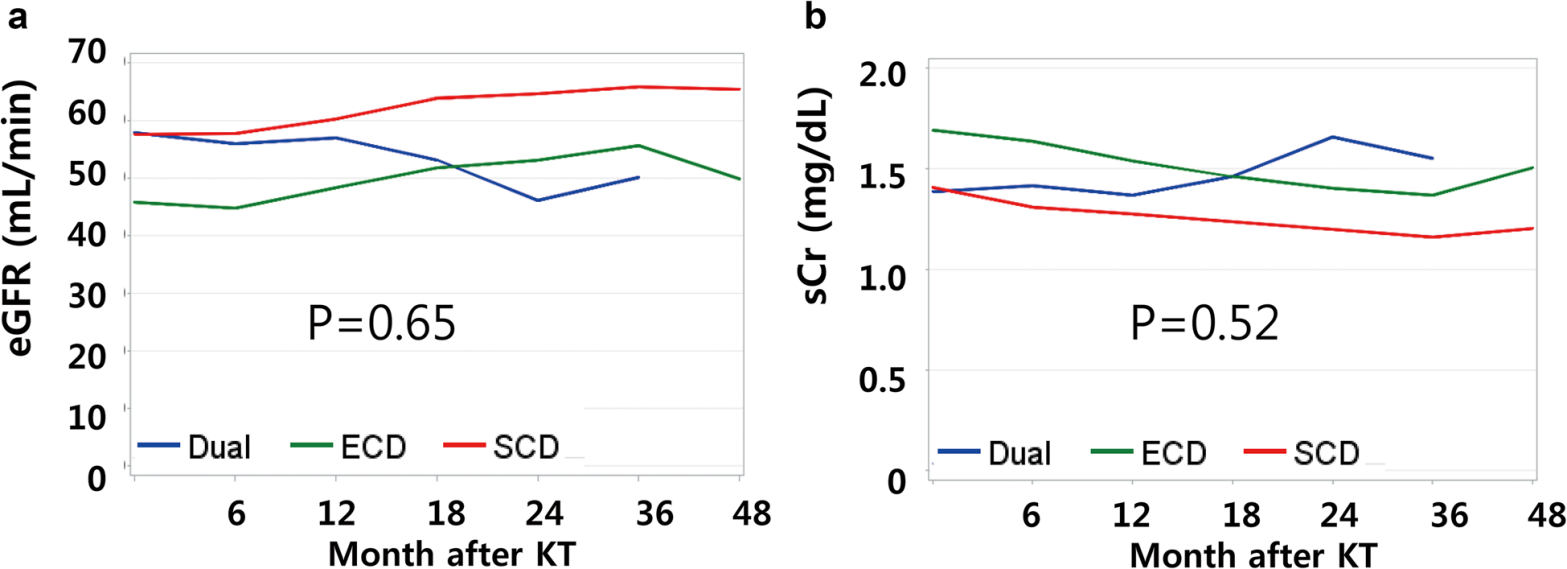
Graft function after kidney transplantation. **a** Post-transplant eGFR at one year after KT was lowest in ECD group. At two and three years after KT, eGFRs were lowest in DKT group. **b** Opposite pattern was seen in sCr level. However, the trend of changing eGFR and sCr level were not significantly different according to each groups

Clinical outcomes are summarized in Table 3. Patient survival rates and death-censored graft survival rates were not different among groups (Fig. 1). Three years after KT, patient survival was 96.2% in the SCD group, 96.2% in the ECD group, and 100% in the DKT group. Death-censored graft survival 3 years after KT was 96.6% in the SCD group, 95.9% in the ECD group, and 100% in the DKT group. There was one graft failure, which occurred in the DKT group. The graft dysfunction was attributed to diabetic nephropathy detected three years after KT, and HD was initiated six months later. Specifically, the recipient had diabetes, but the donor did not. The rate of DGF after DKT (20%) was comparable to that of single SCD KT (26.6%) and was lower than that of single ECD KT (33.8%); however, the differences were not statistically significant (*p* = 0.41). In terms of nadir sCr level and time to nadir sCr, DKT was comparable to single SCD KT and superior to single ECD KT (*p* < 0.01 and 0.02). Post-transplant eGFR one year after KT was lowest in the ECD group (*p* < 0.01). At two and three years after KT, eGFRs were lowest in the DKT group (*p* < 0.01 and 0.01); however, the eGFR and sCr trends were not significantly different among groups (Fig. 2, *p* = 0.65 and 0.52, respectively). The post-operative complication rate after DKT (13.3%) was not higher than that after single ECD KT (23.8%, *p* = 0.99). There was one lymphocele and one ureteral leakage in the DKT group. The lymphocele was controlled after percutaneous drainage, while the ureteral leakage was controlled after double J stent and Foley catheter insertion, which were removed two and four weeks later, respectively.

**Table 3:** Clinical outcomes

	SCD (*n* = 124)	ECD (*n* = 80)	DKT (*n* = 15)	*p* value	SCD vs DKT	ECD vs DKT
Patient death (%)	4 (3.2)	3 (3.8)	0 (0)	0.96		
Graft failure (%)	4 (3.2)	2 (2.5)	1 (6.67)	0.63		
DGF (%)	33 (26.6)	27 (33.8)	3 (20.0)	0.41		
Nadir sCr	1.2 ± 0.7	1.3 ± 0.4	1.0 ± 0.3	< 0.01	0.10	< 0.01
Time to nadir sCr	24.6 ± 30.0	33.8 ± 34.9	22.6 ± 21.3	0.02	0.47	0.04
Post-transplant eGFR						
1 yr	60.2 ± 15.0	48.3 ± 13.9	57.0 ± 21.5	< 0.01	0.58	0.43
2 yr	64.7 ± 18.3	53.1 ± 14.8	46.1 ± 17.4	< 0.01	0.03	0.23
3 yr	65.8 ± 18.0	55.7 ± 17.1	50.1 ± 19.1	0.01	0.15	0.99
Rejection episode	40 (32.3)	31 (38.8)	6 (40.0)	0.59		
Complications^a^ (%)	6 (4.8)	19 (23.8)	2 (13.3)	< 0.01	0.24	0.99
F/U duration (mo)	33.5 ± 15.0	28.5 ± 13.9	25 ± 12.1	< 0.01	0.03	0.39

*DGF* delayed graft function, *sCr* serum creatinine level, *eGFR* estimated glomerulus filtration rate, *F/U* follow up

^a^Complications include ureter leakage, ureter stricture, lymphocele, bleeding, and renal artery stenosis

## Discussion

Outcomes of DKT in our study were not different from those of single KTs in terms of graft survival rate and graft function after KT despite a higher age, higher sCr level, greater burden of diabetes, and higher KDPI and KDRI scores in DKT donors (*p* < 0.01 in all). Disadvantages from the donor factors were overcome by doubling the number of transplanted nephron in DKT. Even though the difference was not statistically significant (*p* = 0.41), the rate of DGF after DKT (20%) was lower than that of single ECD KT (33.8%). It can be explained by that DKT can supply sufficient number of nephron and, even if some fraction of nephrons were injured, enough number of nephrons is preserved to facilitate primary function.

Recently, many studies have reported that graft survival and graft function are not significantly difference between single KT and DKT [5–10, 13–20]. However, the donor selection criteria for DKT among these studies varies. Most studies have used histology based selection criteria such as the 12-point Kalpinski system or the Remuzzi scoring system [5–7, 13–15, 17–19]. In a clinical setting not supported by sufficient pathologists and without a centralized donor management system, scoring of donor kidney biopsy specimens is nearly impossible. Therefore, in our study, we used objective clinical values such as donor age, eGFR, and sCr level as the donor selection criteria for DKT.

KONOS data indicated that the kidney discard rate over the last decade in Koreas was higher among donors aged more than 70 (18.1%) compared to donors younger than 70 (7.4%). Additionally the discard rate in donors between 60 and 70 years was 9.6%. In a clinical setting with insufficient support by pathologists specialized in kidney allograft histology, donor age is the most important factor in the decision of a clinician to discard a kidney graft. Therefore, to reduce the graft discard rate it is necessary to use kidneys from older donors. Numerous studies have reported successful outcomes after DKT from donors older than 70 [7, 14, 16–20]. Thus, we selected 70 as the donor age threshold in our study. The mean donor age in our study was 74.5 yrs. Two donors were younger than 70 (ages 66 and 69), and both had kidneys denied from all other centers.

Even though our results were comparable to the results of single ECD KT, there should be criteria for clinicians to decline a graft kidney. We discarded kidneys when they were grossly discolored or atrophied, or when the donor sCr level had slowly and consistently deteriorated for a period longer than 5 days. Nevertheless, we agree that a histological scoring system is more objective if pathological assessment of the donor kidney is available. Further efforts to establish pathology support systems for kidney graft evaluation are thus necessary.

Our center utilized an “old for old” strategy. As a result, the mean age of recipients in the DKT group (63.7 yrs) was significantly higher than in the ECD group (55.2 yrs., *p* < 0.01). In our study, the eGFR level one year after KT was higher in the DKT group than in the ECD group. However, we found that this situation reversed at two and three years after KT. Although the trend of eGFR change was not significantly different in these groups (*p* = 0.65), it seems reasonable and prudent to transplant kidneys with the potential to last longer into recipients who are expected to live longer. Moreover, the DKT group showed a lower rate of DGF, a lower level of nadir sCr, and a shorter duration to nadir sCr, all of which can lower recipient’s physical burden during the immediate post-operative period. These factors can be helpful, especially for older recipients with less physical reservoir. The duration of dialysis prior to KT was shorter in the DKT group (4.8 years) than in the ECD group (6.1 years), although the difference was not statistically significant (*p* = 0.17). Considering the age of older recipients and the importance of performing KT as soon as possible, DKT should be viewed as a reasonable solution. In addition, Rigotti et al. improved DKT outcomes for older recipients with a personalized immunosuppression strategy [17]. Although we did not utilize this approach, use of mTOR inhibitor to lower doses of calcineurin inhibitors represents a potential way to improve outcomes [17].

There were several limitations to the present study. The number of DKT cases was relatively small and the follow-up duration was too short to draw long-term results. Given these limitations, the ability to generalize our results may be limited. Despite these limitations, this study is valuable because of the successful utilization of kidneys from extremely marginal donors for DKT. To the best of our knowledge, this is the first report of DKT outcomes in an Asian population. Out results also provide evidence for strategies to improve the use of kidneys safely from marginal donors.

## Conclusion

DKT provides comparable graft survival and graft function to single ECD KT despite older donors with higher sCr levels, more diabetes, and higher KDPI and KDRI scores. DKT is a safe and feasible way to use kidney grafts from extremely marginal donors, thereby reducing the organ discard rate. Further efforts should focus on avoiding inappropriate use of unacceptable kidneys and improving DKT outcomes.

## Data Availability

The datasets used and/or analysed during the current study are available from the corresponding author on reasonable request.

## Abbreviations

DD: Deceased donor
DDKT: Deceased donor kidney transplantation
DGF: Delayed graft function
DKT: Dual kidney transplantation
DSA: Donor-specific ant-HLA antibody
ECD: Expanded criteria donor
eGFR: Estimated glomerular filtration
GEE: Generalized Estimating Equation
KDPI: Kidney donor profile index
KDRI: Kidney donor risk index
KONOS: Korean Network for Organ Sharing
KT: Kidney transplantation
PRA: Panel reactive antigen
SCD: Standard criteria donor
sCr: Serum creatinine
UNOS: United Network for Organ Sharing
